# The linguistic validation of the gut feelings questionnaire in three European languages

**DOI:** 10.1186/s12875-017-0626-0

**Published:** 2017-04-20

**Authors:** Marie Barais, Johannes Hauswaldt, Daniel Hausmann, Slawomir Czachowski, Agnieszka Sowinska, Paul Van Royen, Erik Stolper

**Affiliations:** 1 ERCR SPURBO, Department of General Practice, Université de Bretagne Occidentale, Brest, France; 2 Department of General Practice, University Medical Center Göttingen, Göttingen, Germany; 3 Department of Psychology - Applied Social and Health Psychology, University of Zurich, Zurich, Switzerland; 4 Clinical Psychology, Nicolaus Copernicus University, Toruń, Poland; 5 Department of English Studies, Nicolaus Copernicus University, Toruń, Poland; 6 Department of Primary and Interdisciplinary Care, Faculty of Medicine and Health Sciences, University of Antwerp, Antwerp, Belgium; 7 Faculty of Health, Medicine and Life Sciences, Caphri School for Public Health and Primary Care, Department of Family Medicine, Maastricht University, Maastricht, The Netherlands

## Abstract

**Background:**

Physicians’ clinical decision-making may be influenced by non‐analytical thinking, especially when perceiving uncertainty. Incidental gut feelings in general practice have been described, namely, as “a sense of alarm” and “a sense of reassurance”.

A Dutch Gut Feelings Questionnaire (GFQ) was developed, validated and afterwards translated into English following a linguistic validation procedure.

The aims were to translate the GFQ from English into French, German and Polish; to describe uniform elements as well as differences and difficulties in the linguistic validation processes; to propose a procedural scheme for future GFQ translations into other languages.

**Methods:**

We followed a structured, similar and equivalent procedure. Forward and backward-translations, repeated consensus procedures and cultural validations performed in six steps. Exchanges between the several research teams, the authors of the Dutch GFQ, and the translators involved continued throughout the procedure.

**Results:**

12 translators, 52 GPs and 8 researchers in the field participated to the study in France, Germany, Switzerland and Poland. The collaborating research teams created three versions of the 10-item GFQ. Each research team found and agreed on compromises between comparability and similarity on one hand, and linguistic and cultural specificities on the other.

**Conclusions:**

The gut feeling questionnaire is now available in five European languages: Dutch, English, French, German and Polish. The uniform procedural validation scheme presented, and agreed upon by the teams, can be used for the translation of the GFQ into other languages. Comparing results of research into the predictive value of gut feelings and into the significance of the main determinants in five European countries is now possible.

**Electronic supplementary material:**

The online version of this article (doi:10.1186/s12875-017-0626-0) contains supplementary material, which is available to authorized users.

## Background

Physicians’ clinical decision-making is based on the interaction of analytical and non-analytical reasoning and gut feelings can be considered a part of the non-analytical reasoning process [1]. In 2009, the concept of gut feelings in general practice was described, by means of a qualitative study, as a sense of alarm and a sense of reassurance [2]. The sense of alarm is “an uncomfortable feeling experienced by the physician, that something does not fit in a patient’s clinical presentation although he/she has found no specific indications”. The sense of alarm “activates the diagnostic process and induces the doctor to initiate specific management to prevent serious health problems” [3]. The sense of reassurance means that a GP “feels secure about the further management and course of a patient’s problem, even though he/she may not be certain about the diagnosis: “everything fits in” [3]. Gut feelings are considered to play a substantial role in the diagnostic reasoning of GPs [1]. Two prospective studies proved how this sense of alarm could be efficient. When dealing with children with serious infections, GPs’ gut feeling about parental concerns and the children’s appearance had a high specificity and a high positive likelihood ratio [4]. Gut feelings that something was wrong were also a common reason for referral which proved to be a strong predictor of cancer in a Danish cancer pathway [5].

A Dutch Gut Feelings Questionnaire (GFQ) was created from the consensus criteria for gut feelings and validated by a construct validation procedure using case vignettes [6]. The validity of the GFQ was consistent: the internal consistency of the GFQ proved to be high (Cronbach’s alpha = 0.91), the Kappa with quadratic weighting was moderate to good (0.62, 95% CI: 0.55-0.69) and factor analysis showed one factor with opposites for sense of reassurance and sense of alarm items. Two versions of the questionnaire were created: a vignette version and a real case version. A linguistic validation procedure was performed to obtain an English version of the questionnaire in general practice [6].

The aim of this article is to report on the translation procedure of the GFQ from English into French, German and Polish; to describe uniform elements as well as differences and difficulties in the linguistic validation processes; to propose a procedural scheme for future GFQ translations into other languages.

## Methods

Research teams are composed of French, German, Swiss, and Polish speaking researchers in different countries in primary care.

The linguistic validation procedure which the teams followed met the standardisation criteria found in the international literature [7–11]. It was in line with the way researchers had translated the Dutch questionnaire into English [6].

**Fig. 1:**
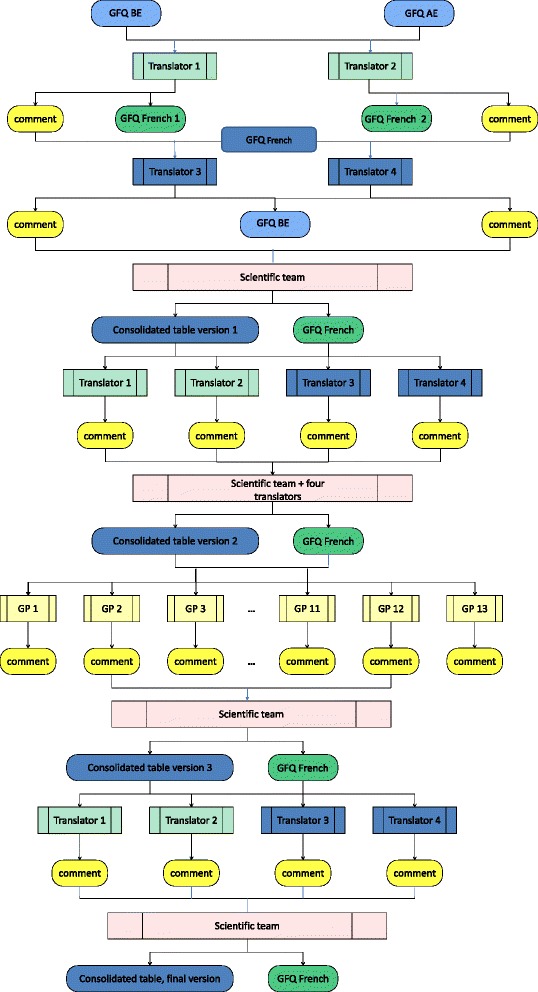
The procedural scheme followed for the English-French translation of the gut feelings questionnaire. GFQ: Gut Feelings Questionnaire, BE: British English, AE: American English

The linguistic validation process consisted of six steps: Forward-translation (step 1), backward-translation (step 2), first consensus (step 3), cultural validation (step 4), second consensus (step 5), and final version (step 6). Table 1 provides a summary of the different steps in all three versions and Fig. 1 provides the procedural scheme followed.

**Table 1:** A summary of the different steps of the linguistic and cultural validation

6 steps
• Step n°1 = separate and independent forward translation by two native speakers into the intended language
• Step n°2 = separate and independent backward translation of the two results as obtained from step n°1 by two English native speakers.
• Step n°3 = first consensus version of the questionnaire obtained after comparison of the versions resulting from step N°1 and 2 by the research team.
• Step n°3 = first consensus version of the questionnaire obtained after comparison of the versions resulting from step N°1 and 2 by the research team.
• Step n°5 = second consensus with the summary of the GPs’ comments and suggestions for modifications submitted to the four translators and the research team.
• Step n°6 = last consensus and definitive version of the questionnaire in the intended language.

We have obtained the approval of the ethics committee of the University de Bretagne Occidentale for the study (N°05092012). Informed consent was obtained from all participants even thoughit was a non-interventional study.

### Forward-backward translations (step 1 and 2)

Two native-speaking translators for each language (French, German and Polish) who were familiar with medical terms, translated the questionnaire into their own language. They performed this translation separately and independently after receiving information about the goal of the questionnaire and the way it would be used in research. They were invited to add comments if needed (step 1).

Next, two native-speaking English language translators, familiar with medical terms, provided independently and separately two backward-translations, each using a different forward-translated version. They were also invited to add comments if needed (step 2).

### Reaching a first consensus (step 3)

Each research team prepared a first draft for a consensus translation in their own language, putting all the differences and questions in an extended table. The four translators, each belonging to the French, German or Polish groups, were separately asked to read this first consensus carefully, including all the comments in the table, and to add their opinions to this table. Afterwards, each research group adjusted the consensus and collected all the remaining questions and translation problems in a new table. A meeting was then arranged, with all four translators, in which undecided items were discussed.

Extensive communication between the translators, the coordinating scientific team, and the authors of the original Dutch version yielded a consensual GFQ version, in each language: French, German and Polish.

### Cultural validation (step 4)

These consolidated GFQ versions were sent to at least ten GPs (native speakers of French, German or Polish) based in France, Germany, Switzerland or Poland, asking them to check for grammatical errors and cultural misunderstandings. An accompanying letter explained the background of the GFQ and the purpose of their involvement.

### Reaching a second consensus (step 5)

The results of the GPs’ feedback were incorporated into an advanced version of GFQ by the research team. All previous stakeholders in this process and interested parties added some comments which were integrated. The four translators studied the comments and gave their final judgment.

### Resulting in a final version (step 6)

After considering the translators’ recommendations, each research group finally determined the definitive text of the questionnaire.

## Results

A French, German and Polish version of the English GFQ version is now available. Table 2 provides the GFQ in the four languages: the original in English, along with the French, German and Polish versions.

**Table 2:** Four versions of the gut feeling questionnaire

English	French	German	Polish
1. I feel confident about my management plan and/or about the outcome: it all adds up.	1. J’ai confiance dans la prise en charge que je propose et/ou dans ses résultats attendus : tout est cohérent.	1. Ich fühle mich sicher in Bezug auf meinen Behandlungsplan und/oder das klinische Ergebnis: Es passt alles gut zusammen.	1. Jestem pewny co do mojego planu postępowania i/lub wyników: wszystko zgadza się.
2. I am concerned about this patient’s state of health: something does not add up here.	2. Je suis préoccupé(e) par l’état de santé de ce patient : quelque chose ne va pas.	2. Ich bin besorgt über den Gesundheitszustand dieses Patienten: hier stimmt etwas nicht.	2. Jestem zaniepokojony stanem zdrowia tego pacjenta: coś tu się nie zgadza.
3. In this particular case, I will formulate provisional hypotheses with potentially serious outcomes and weigh them against each other.	3. Pour ce cas précis, je vais formuler des hypothèses de pathologies potentiellement graves que je confronterai les unes aux autres.	3. In diesem speziellen Fall werde ich vorläufige Verdachtsdiagnosen formulieren, mit möglicherweise schwerwiegenden Folgen, die ich gegeneinander abwägen muss.	3. W tym konkretnym przypadku sformułuję tymczasowe hipotezy z potencjalnie istotnymi wynikami i porównam je.
4. I have an uneasy feeling because I am worried about potentially unfavourable outcomes.	4. Je suis gêné (e) parce que je redoute de possibles conséquences graves pour ce patient.	4. Ich habe ein ungutes Gefühl, weil ich über mögliche ungünstige Folgen besorgt bin.	4. Mam niejasne przeczucie ponieważ martwią mnie potencjalnie niekorzystne wyniki.
5. This case requires specific management to prevent any further serious health problems.	5. Ce cas nécessite une prise en charge spécifique afin d’éviter d’autres problèmes de santé graves pour le patient.	5. Dieser Fall erfordert eine besondere Herangehensweise, um mögliche ernste Komplikationen zu vermeiden.	5. Ten przypadek wymaga szczególnego postępowania aby zapobiec dalszym poważnym problemom zdrowotnym.
6. What course of action have you chosen? (Please tick one answer) I will … - Wait and see. - Not take action, but will invite the patient for a follow‐up appointment either face‐to‐face or by phone. - Arrange further testing (laboratory tests, X‐rays, etc.). - Arrange further testing, and in the meantime, I will start treatment (medicinal or other). - Start treatment, but will not arrange a follow‐up. - Start treatment and will invite the patient for a follow‐up appointment either face‐to‐face or by phone. - Refer the patient.	6. Quel plan d’action avez-vous choisi (une seule réponse possible). J’ai décidé : -D’attendre, de temporiser. -De ne pas prendre de décision pour le moment et de proposer au patient un rendez-vous de suivi au cabinet ou par téléphone. - De programmer des examens complémentaires (analyses au laboratoire, radiographies, etc.…). - De programmer des examens complémentaires et de mettre sans attendre le patient sous traitement (médicamenteux ou autre). - De démarrer un traitement sans organiser de suivi. - De démarrer un traitement et de proposer au patient un rendez-vous de suivi, au cabinet ou par téléphone. -D’adresser le patient vers un spécialiste en urgences ou non.	6. Wie sieht Ihr weiteres Vorgehen aus? (Bitte nur eine Antwort ankreuzen.) Ich werde… - die Situation abwartend offenhalten. - jetzt noch nichts unternehmen, aber den Patienten zu einem persönlichen oder telefonischen Kontrolltermin bitten. - weitere Untersuchungen veranlassen (Labortest, Röntgenbild, etc.). - weitere Untersuchungen veranlassen, in der Zwischenzeit aber bereits die Behandlung beginnen (medikamentös oder anderes). - mit der Behandlung beginnen, aber keinen Kontrolltermin vereinbaren. - mit der Behandlung beginnen, und den Patienten zu einem persönlichen oder telefonischen Kontrolltermin bitten. - den Patienten überweisen.	6. Jaki rodzaj postępowania wybrałeś? (zaznacz jedną odpowiedź) -Poczekam i zobaczę jak się sytuacja rozwinie. - Nie podejmę jeszcze działania, ale umówię się z pacjentem na wizytę kontrolną w gabinecie lub na konsultację telefoniczną. - Zlecę dalsze badania (badania laboratoryjne, RTG, itd.). - Zlecę dalsze badania a w międzyczasie rozpocznę leczenie (leki lub inny rodzaj postępowania). - Rozpocznę leczenie bez umawiania. - Rozpocznę leczenie i umówię pacjenta na wizyty kontrolne w gabinecie lub na konsultację telefoniczną. - Skieruję pacjenta gdzieś indziej.
7. This patient’s situation gives me reason to arrange a follow‐up visit sooner than usual or to refer him or her more quickly than usual to a specialist.	7. L’état de santé de ce patient impose une visite de surveillance plus tôt que prévu, ou que le patient soit dirigé plus tôt que prévu vers un spécialiste.	7. Die Situation dieses Patienten veranlasst mich, den nächsten Konsultationstermin früher als üblich zu vereinbaren oder ihn rascher als sonst an einen Spezialisten zu überweisen.	7. Sytuacja pacjenta daje mi podstawy aby umówić go na wizytę kontrolną wcześniej niż zwykle lub skierować jego lub ją do specjalisty szybciej niż zwykle.
8A. What do you consider to be the most likely diagnosis? (Please tick one answer.) -My most likely diagnosis is …. - There are several possible diagnoses; − I am unable to choose one at this moment. 8B. And which diagnosis will determine your management?…	8A. Quel est selon vous le diagnostic le plus probable ? (une seule réponse possible) -Pour moi le diagnostic le plus probable est … -Je ne suis pas en mesure de me prononcer pour le moment. 8B. Quel diagnostic va déterminer votre prise en charge ? …	8A. Was ist Ihrer Ansicht nach die zutreffendste Diagnose? (Bitte nur eine Antwort ankreuzen.) - Meine zutreffendste Diagnose ist… - Es gibt mehrere mögliche Diagnosen; zum jetzigen Zeitpunkt kann ich keine wählen. 8B. Und welche Diagnose bestimmt Ihren Behandlungsplan?…	8A. Jaka według Ciebie diagnoza jest najbardziej prawdopodobna? (Proszę zaznaczyć jedną odpowiedź). - Najbardziej prawdopodobną diagnozą według mnie jest… - Istnieje kilka możliwych rozpoznań; nie jestem w stanie w tym momencie wybrać jednego z nich. 8B. Która diagnoza w takim razie zdecyduje o Twoim postępowaniu?…
9. How confident are you in the diagnosis that you indicated under 8b as determining your management? ____%	9. Quel degré de certitude accordez-vous au diagnostic inscrit pour la réponse 8B ? Je suis sûr(e) à _____%	9. Wie sicher sind Sie sich bei der Diagnose, die Sie bei Frage 8b als ausschlaggebend für Ihren Behandlungsplan angegeben haben? ____%	9. Na ile jesteś pewny tej diagnozy, którą wskazałeś w punkcie 8b jako decydującą o Twoim postępowaniu?____%
10. Please indicate what kind of gut feeling you have at the end of the consultation: -Something is wrong with this picture. -Everything fits. -Impossible to say, or not applicable.	10. Décrivez votre ressenti à la fin de la consultation : -Il y a quelque chose qui cloche -Tout se tient -Je n’ai pas d’avis ou ce n’est pas applicable à cette situation.	10. Bitte beschreiben Sie Ihr Bauchgefühl am Ende des Beratungsgesprächs: - Hier stimmt etwas nicht. - Alles passt zusammen. - Kann ich unmöglich sagen, oder trifft nicht zu.	10. Proszę określić jaki rodzaj przeczucia występuje u Ciebie pod koniec konsultacji: - Wydaje się, że nie wszystko tutaj jest w porządku. - Wszystko pasuje. - Nie da się stwierdzić albo nie dotyczy.

### French procedure: adaptations and problems

These six steps were completed between October 2012 and May 2013.

#### Step 1 to 3

We only translated the real case questionnaire in the French procedure because we intended to use it for a study in real settings and had no research proposal related to the vignettes questionnaire.

Three translators were from the linguistic department of the University of Brest: two French native speakers and a British English native speaker. The fourth was a French GP whose native language is British English. The scientific team was composed of one GP trainee, working on a gut feelings master’s thesis, and two members of the department of General Practice working on the same topic.

Several points needed to be discussed for the French translation:

For the fourth item: “I have an uneasy feeling because I am worried about potentially unfavourable outcomes,” the proposition “I have an uneasy feeling” was translated as “Je suis gêné” “I am bothered”. The phrasing “uneasy feeling” was not compatible in the French version.

For the sixth item: “What course of action have you chosen? (Please tick one answer.) I will wait and see”, the concept of “wait and see” does not exist in the French language, and this expression is also used verbatim in English. We chose to translate it as “attendre, temporiser”: “to wait, to temporise,” meaning staying open to new things which could happen.

#### Step 4 to 6

We submitted the translated questionnaire to 12 GPs who were experienced in research in primary care. We analysed the 12 answers we received.

For the first item: “I feel confident about my management plan and/or about the outcome: it all adds up”, 7 participants did not understand the proposition “about the outcome”: they found it difficult to make such a judgement at this early stage of the diagnostic reasoning process. They asked about the kind of outcome: the expected outcome or the actual outcome, and the outcome of the management plan and the tests requested or the outcome of the treatment plan. The participants’ lack of understanding was related to discomfort with the clinical reasoning process at an early stage in the case and not with the terminology. We chose to add “expected outcome” to the first proposition.

Seven participants wanted to add the referral to the emergency unit to item n° 6: “What course of action have you chosen?” “Refer the patient”. For French GPs, referring to the emergency unit or to the specialist are two different situations. To the authors of the original Dutch version, the idea was to include the referral, not distinguishing between urgent and non-urgent. We maintained the original meaning of item 6 and added the following details: “refer the patient to a specialist, either within the emergency unit or elsewhere.”

For the seventh item: “This patient’s situation gives me reason to arrange a follow‐up visit sooner than usual or to refer him or her more quickly than usual to a specialist”, 5 participants asked that the wording “sooner than usual” be defined more precisely. They found the “usual” situation difficult to define. For the authors of the original Dutch version, “sooner than under usual care” means “sooner than he/she does in common daily situations, without hurrying”. “To refer him or her more quickly than usual to a specialist” was also confusing for these 5 participants. They asked that “or to the emergency unit” be added. As for the sixth item, in accordance with the authors of the original Dutch version, we chose to maintain the generic term “to the specialist” without mentioning the emergency unit.

The French version of the English GFQ version is available (See Additional file 1).

### German procedure: adaptations and problems

The six steps were completed between April 2014 and June 2015.

#### Steps 1 and 2

We translated both the real practice and the case vignette design from the BE version.

As the German language varies somewhat between regions and countries, we intended to find a supranational linguistic German version. Therefore translators, and members of the scientific team involved, were drawn from different countries and regions, e.g., Germany (D) (northern and southern regions) and Switzerland (CH).

All translators were from different institutions and lived and worked in Germany, Switzerland, the United Kingdom, or the United States of America. The research team was composed of one general practitioner with academic background from Germany (JH) and one University psychologist from Switzerland (DH), both doing academic research in the field of intuition and medical decision-making.

#### Step 3

Our actual execution of step 3 differed slightly from the adopted procedural scheme in three ways: first, by performing an intermediate step with two additional leading versions; second, by subsequently communicating by multiple e-mail exchanges and/or short physical meetings (instead of holding a telephone meeting), and third, by continuously involving the original Dutch authors (in particular ES).

As an extra intermediate step, DH and JH independently proposed two leading versions as summaries of the four heterogeneous versions and comments of all the translators. Then a first consensus was reached between DH and JH, based on all the existing versions and comments, which tended to favour one of the proposed leading versions chosen by the preparation team. The consolidated table, including all versions and comments, was then sent to all the translators and the whole research team for further revision or comments. Another advantage of proposing two additional leading versions has been that a telephone conference involving everyone was not necessary. DH and JH had a meeting at the end of step three with the aim of checking, discussing and integrating the final comments, and planning further steps (e.g., cultural validation).

#### Step 4 to 6

Twenty physicians (mainly GPs) had been asked in February 2015 to do a cultural check of the penultimate version. Subsequently, 12 questionnaires from responders were systematically analysed, comment by comment, by the preparation team. Items 1, 3, and 8b were discussed by the research team in detail at a second meeting (in March 2015). The team voted to maintain the status quo, whereas items 4: “I have an uneasy feeling because I am worried about potentially unfavourable outcomes” (reformulated as: “weil ich… besorgt bin”) and 6: “What course of action have you chosen? I will Wait and see” (first option added with: “die Situation abwartend offenhalten” which means stay open to what could happen) have been slightly adjusted. “Abwartendes Offenhalten” in GP semantics is the German equivalent to the English “watchful waiting” (“wait and see”), and has always to be weighed against “abwendbar gefährlicher Verlauf” (preventable dangerous outcome).

In an additional step, we asked for final comments from all significantly involved participants, including the whole research team and all the translators.

Item 8a: “What do you consider to be the most likely diagnosis?” caused doubt until the very end of the German linguistic validation process. For this item, the following suggestions had been under consideration, with subtly different meanings: “die wahrscheinlichste” (the most likely) (also used by medics in the UK), “die bevorzugte” (the preferred), or “die zutreffendste” (the most appropriate). Finally, the following wording was chosen: “Was ist Ihrer Ansicht nach die zutreffendste Diagnose? Meine zutreffendste Diagnose ist…” in the sense of the most appropriate diagnosis.

The final German versions were called “Fragebogen zum Bauchgefühl bei ärztlichen Entscheidungen” (FBAE). Generally, the English case vignette design and the real practice version differed very little. The subtle differences in the German version can be found in items 6, 7, and 8b in the word “würde” (instead of “werde”), and in item 8a in the words “wäre” (instead of “ist”) and “könnte” (instead of “kann”).

The German version of the English GFQ version is available (See Additional file 2).

### Polish procedure: adaptations and problems

#### Step 1 to 3

We translated both the real practice and case vignettes from the BE version into Polish.

All the translators were affiliated to different academic institutions and all had a linguistic background. There were two Polish certified translators with expertise in medical translation, one translator from the English Department of Nicolaus Copernicus University, and one American native speaker. The research team was composed of one general practitioner and a linguist, both from Nicolaus Copernicus University.

The problem that occurred at this stage involved the translation of Items 6 and 7 and was due to cultural differences: “This patient’s situation gives me reason to arrange a follow‐up visit sooner than usual or to refer him or her more quickly than usual to a specialist.” First of all, phone consultations are not commonplace in Poland. GPs have no obligation to call their patients to arrange visits. Secondly, referring the patient more quickly than usual to a specialist is not possible at all in Polish primary care due to one national medical service provider which controls and manages the whole referral system. Yet, after discussion, we decided to include these items as they are present in the English version and proceeded to the next step. Finally, the translation of “would refer the patient” as “odesłałbym pacjenta” has negative connotations in Polish and implies ignoring and sending away the patient. For that reason, after consultation with the translators, we came up with a neutral expression “skierowałbym pacjenta gdzieś indziej.” (I would refer the patient somewhere else), which communicates the meaning of sending a patient to someone else, rather than getting rid of the patient as it is the case with “odsyłać” in Polish.

#### Steps 4 to 6

We sent the translated questionnaire to 25 GPs via email and asked for a cultural check and evaluation of the equivalence between the translations and the BE versions. Two e-mail addresses turned out to be incorrect and, out of 23 GPs, only eight GPs with an academic background and experience in research in primary care responded. All of them evaluated the translations positively (real practice and case vignette). Four of the GPs provided constructive comments and feedback. The proposed linguistic corrections concerned Items 1, 3, 7 and 10. These items were thoroughly discussed by the scientific team and consensus was reached.

In Item 1: “I feel confident about my management plan and/or about the outcome: it all adds up,” there is no Polish adequate expression for “it all adds up.” The closest expression: “wszystko składa się w jedną całość” was rejected and replaced with “wszystko zgadza się,” (everything is fine) which is more comprehensible and more common in professional language among GPs.

In Item 3: “In this particular case, I will formulate provisional hypotheses with potentially serious outcomes and weigh them against each other,” the phrase: “rozważę ich wzajemne związki” for “weigh them against each other” was replaced with “porównam je,” (compare them) which more adequately renders the original concept and simplifies the translation. At the same time, the respondents found it more comprehensible than the previous choice.

In Item 7:” This patient’s situation gives me reason to arrange a follow‐up visit sooner than usual or to refer him or her more quickly than usual to a specialist,” the phrase: “Obecny stan zdrowia pacjenta,” which means „the patient’s health condition” was replaced with „sytuacja pacjenta,” which sits better within the holistic model adopted in general practice. It is the medical term used by GPs as it expresses not only a patient’s somatic condition, but also his or her psycho-social condition.

In Item 10: “Please indicate what kind of gut feeling you have at the end of the consultation,” the word “Intuicja” (“intuition”) has been replaced with “przeczucie” (“gut feeling”), which is more appropriate in the everyday language of general practice.

The Polish version of the English GFQ version is available (See Additional file 3). The English version of the GFQ is available (See Additional file 4).

## Discussion

### Main findings

The GFQ has been translated into three more European languages using a standardised procedure of linguistic validation. The collaborating research teams from France, Germany/Switzerland and Poland found and agreed on compromises between comparability and similarity on one hand, and linguistic and cultural specificities on the other. All the GFQ versions are available on the website http://www.gutfeelings.eu.

### Strengths and limitations of the study

Translators with a medical background worked on the questionnaire following the standardised procedure. This feature was important here to avoid misunderstandings in the specific area of medical decision-making. The cultural check stage was undertaken with GPs who were the principal recipients of the questionnaire. They gave a pragmatic point of view as they are active in the field of daily clinical practice.

The French, German and Polish teams were working in the same research network on clinical decision- making. The creators of the questionnaire were involved from the beginning of the process and acted as the vital link between the researchers. These two characteristics facilitated exchanges and probably prevented the translation from deviating from the original Dutch version of the questionnaire.

Similar items generated discussions in the three different research teams. Expressions such as “uneasy feeling” and “wait and see” do not correspond to existing linguistic concepts in French, German or Polish but may be reflected in analogy, at least in German, by “Alarmgefühl” and “abwartendes Offenhalten”.

### Comparing with existing literature

As far as we know, the GFQ is the first tool developed which measures GPs’ gut feelings. There is no alternative tool available at present. The sense of alarm was recognised by European GPs in their daily practice [12]. The transculturality of the gut feelings concept between Proto-Germanic and Romance languages was revealed after a Delphi procedure compared the Dutch and the French statements of the gut feelings criteria [13]. German research into this field had been sparked in 2004 by the Dutch expression “niet pluis” literally “there is danger here, something is amiss” which is commonplace for Dutch GPs but has no equivalent in German, although German GPs also expressed their incidental uneasiness (“Hier stimmt’was nicht!”) which was later coined as “Alarmgefühl”. The French and German versions of the questionnaire logically followed this finding. The linguistic validation procedures followed here, in Polish, allowed us to expand the concept to include Slavic languages. We assume that the utility of the GFQ would also be transferable, working within this transcultural context and applying standardised linguistic procedures. The forward- backward translation, with cultural check, was preferred here to the Delphi procedure [14]. Exchanges between several translators with a medical background, GPs and a linguist allowed us to analyse in depth differences in wording.

The Dutch first authors on the gut feelings concept had an idiomatic expression in their language to express the sense of reassurance and the sense of alarm “pluis/niet pluis”. A survey in 2005 identified idiomatic expressions in European languages about this specific term “gut feeling” [12]. Even if no specific expression existed to describe this feeling, European GPs recognised the description of the sense of alarm. Behind the linguistic aspects, GPs do share the same medical decision-making model. A consultation in general practice is complex: the patient may suffer from non-specific symptoms; he will use his own words and the GP has to translate into semiological language. The clinical signs are partial and rarely discriminative. Few tests are available at the surgery to support his hypotheses. The stress of dealing with a potentially severe disease, as well as time management, complicate the task of the practitioner. The GP has to make a decision in this uncertain and incomplete area [15, 16]. Two different interacting modes which control the activity of reasoning were described: the intuitive mode or system 1 and the analytical mode or system 2 [17]. The analytical mode operates consciously; it is selective and limited in resources, slow, laborious and sequential. It is a very powerful system because of its important computing capacity but it is difficult to sustain over a long period. The intuitive mode has opposite characteristics: it operates unconsciously, it is unlimited, works fast and automatically. It considers several actions concurrently. It connects similar elements with previous situations and activates stored rules. This dual process theory is now integrated into clinical reasoning and the medical educational process [17–19]. The sense of alarm is recognised here as a feedback mechanism, compelling the physician to abandon his routine-based/schematic mode of reasoning in favour of an analytical and attentional one [20, 21].

Whilst the organisation of health care systems in The Netherlands and Belgium, where the original version was validated, are similar, the health care systems in France, Germany, Switzerland and Poland are organised differently in terms of structure, process and outcome [22–24]. The application of medical decisions has to integrate into each different type of organisation. The GFQ was modified to correspond to French, Polish and German systems. French GPs distinguished between referral to a specialist and referral to the emergency unit. In the first case, they sought the opinion of a specialist within their own network to obtain a second point of view of the patient, with non-formal emergency criteria. When they referred to the emergency unit, they needed to seek a second opinion with urgent and appropriate care. We kept the original version of the questionnaire, with additions, on this specific point in the French questionnaire. In Poland the same item was problematic because of the national medical service provider which controls the referral system. Polish authors found a neutral formulation to express the sense of the proposition without insisting on the organisational aspect. No adaptations were needed in the German version: German and Swiss GPs did understand each proposition in the original formulation during the cultural validation. Their health care system is closer to the Dutch one on this particular point.

### Implications for practice and future research

Translating the GFQ into different languages using a standardised procedure is of great value for further quantitative research. A study protocol has been designed to evaluate the feasibility of the questionnaire in daily practice in primary care. A quantitative phase will explore the average time taken to fill in the questionnaire, estimated by the GP, the disruption of daily routine caused by the gut feelings questionnaire with a four-point scale, and additional workload created by completing the questionnaire with a four-point scale. A qualitative phase, using semi-structured interviews with the GPs involved, will explore the integration of the questionnaire into daily practice.

The accuracy of gut feelings is another point to be studied. A prospective observational study, using the GFQ to measure the accuracy of the general practitioner’s sense of alarm when confronted with dyspnoea and/or thoracic pain, is actually planned [25].

The GFQ may also be useful in the field of education. Gut feelings appeared in tutorial dialogue between Dutch trainees and their supervisors [26]. When they faced uncertainty during consultation, trainees had to take their gut feelings into account during the reasoning process [26]. We visualise the GFQ as a tool which will facilitate the explanation of how non-analytical reasoning forms part of the teaching of clinical decision-making [27, 28]. A think aloud study is also planned, to check the way GPs understand each item when dealing with case vignettes. Manipulating cues in case vignettes and measuring their influence on the results of the GFQ may be an interesting possibility. Modifications to the GFQ may occur in the future due to the integration of the results of new studies.

## Conclusions

The gut feeling questionnaire is now available in five European languages: Dutch, English, French, German and Polish. The uniform procedural scheme presented, which the teams agreed on, can be used for the translation of the GFQ into other European languages. Comparing results of research into the predictive value of gut feelings in several European countries, where the native language is one of these five, is now possible.

## Additional files

Additional file 1: GFQ French Version. The French version of the Gut Feeling Questionnaire. (DOCX 29 kb)

Additional file 2: GFQ German Version. The German version of the Gut Feeling Questionnaire. (DOCX 30 kb)

Additional file 3: GFQ Polish Version. The Polish version of the Gut Feeling Questionnaire. (DOCX 29 kb)

Additional file 4: GFQ English version. The English version of the Gut Feeling Questionnaire. (DOCX 27 kb)

## Abbreviations

GFQ: Gut feeling questionnaire
GP: General practitioner
