# Supplementary material for: The linguistic validation of the gut feelings questionnaire in three European languages
Source: BMC Fam Pract. 2017 Apr 20;18:54. doi: 10.1186/s12875-017-0626-0 (PMC5437565; doi:10.1186/s12875-017-0626-0)
Supplement: Supplementary file 1 — GFQ French Version. The French version of the Gut Feeling Questionnaire. (DOCX 29 kb) [file 12875_2017_626_MOESM1_ESM.docx]

Pas du tout d’accord

Pas d’accord

Sans opinion

D’accord

Tout à fait d’accord

1. J’ai confiance dans la prise en charge que je propose

et / ou dans ses résultats attendus : tout est cohérent. ❑ ❑ ❑ ❑ ❑

1. Je suis préoccupé(e) par l’état de santé de ce patient :

quelque chose ne va pas. ❑ ❑ ❑ ❑ ❑

1. Pour ce cas précis, je vais formuler des hypothèses de

pathologies potentiellement graves que je confronterai

les unes aux autres. ❑ ❑ ❑ ❑ ❑

1. Je suis gêné (e) parce que je redoute de possibles

conséquences graves pour ce patient. ❑ ❑ ❑ ❑ ❑

1. Ce cas nécessite une prise en charge spécifique afin

d’éviter d’autres problèmes de santé graves pour le patient. ❑ ❑ ❑ ❑ ❑

1. Quel plan d’action avez-vous choisi (une seule réponse possible). J’ai décidé :

❑ D’attendre, de temporiser.

❑ De ne pas prendre de décision pour le moment et de proposer au patient un rendez-vous de suivi au cabinet ou par téléphone.

❑ De programmer des examens complémentaires (analyses au laboratoire, radiographies, etc…).

❑ De programmer des examens complémentaires et de mettre sans attendre le patient sous traitement (médicamenteux ou autre).

❑ De démarrer un traitement sans organiser de suivi.

❑ De démarrer un traitement et de proposer au patient un rendez-vous de suivi, au cabinet ou par téléphone.

❑ D’adresser le patient vers un spécialiste ou vers les urgences.

1. L’état de santé de ce patient impose une visite de surveillance rapidement, ou que le patient soit dirigé plus tôt que rapidement vers un spécialiste ou vers les urgences.

❑ Oui ❑ Non

1. A. Quel est selon vous le diagnostic le plus probable ? (une seule réponse possible)

- Pour moi le diagnostic le plus probable est …………………………
- Je ne suis pas en mesure de me prononcer pour le moment.

B. Quelle hypothèse diagnostique va déterminer votre prise en charge?

…………………………………………………………………………………….

1. Quel degré de certitude accordez-vous au diagnostic inscrit pour la réponse 8B ?

Je suis sûr(e) à ……..%

1. Décrivez votre ressenti à la fin de la consultation :

❑ Il y a quelque chose qui cloche

❑ Tout se tient

❑ Je n’ai pas d’avis ou ce n’est pas applicable à cette situation
