# Supplementary material for: The linguistic validation of the gut feelings questionnaire in three European languages
Source: BMC Fam Pract. 2017 Apr 20;18:54. doi: 10.1186/s12875-017-0626-0 (PMC5437565; doi:10.1186/s12875-017-0626-0)
Supplement: Supplementary file 2 — GFQ German Version. The German version of the Gut Feeling Questionnaire. (DOCX 30 kb) [file 12875_2017_626_MOESM2_ESM.docx]

Stimme ganz und gar nicht zu

Stimmer eher nicht zu

Unentschieden

Stimme eher zu

Stimme voll und ganz zu

1. Ich fühle mich sicher in Bezug auf meinen Behandlungsplan und/oder das klinische Ergebnis: Es passt alles gut zusammen ❑ ❑ ❑ ❑ ❑
2. Ich bin besorgt über den Gesundheitszustand dieses Patienten:

hier stimmt etwas nicht. ❑ ❑ ❑ ❑ ❑

1. In diesem speziellen Fall werde ich vorläufige Verdachtsdiagnosen

formulieren, mit möglicherweise schwerwiegenden Folgen,

die ich gegeneinander abwägen muss. ❑ ❑ ❑ ❑ ❑

1. Ich habe ein ungutes Gefühl, weil ich über mögliche

ungünstige Folgen besorgt bin. ❑ ❑ ❑ ❑ ❑

1. Dieser Fall erfordert eine besondere Herangehensweise,

um mögliche ernste Komplikationen zu vermeiden. ❑ ❑ ❑ ❑ ❑

1. Wie sieht Ihr weiteres Vorgehen aus? (Bitte nur eine Antwort ankreuzen.) Ich werde…

❑ Die Situation abwartend offenhalten.

❑ Jetzt noch nichts unternehmen, aber den Patienten zu einem persönlichen oder telefonischen Kontrolltermin bitten.

❑ Weitere Untersuchungen veranlassen (Labortest, Röntgenbild, etc.).

❑ Weitere Untersuchungen veranlassen, in der Zwischenzeit aber bereits die Behandlung beginnen (medikamentös oder anderes).

❑ Mit der Behandlung beginnen, aber keinen Kontrolltermin vereinbaren.

❑ Mit der Behandlung beginnen, und den Patienten zu einem persönlichen oder telefonischen Kontrolltermin bitten.

❑ Den Patienten überweisen.Quel plan d’action avez-vous choisi (une seule réponse possible).

1. Die Situation dieses Patienten veranlasst mich, den nächsten Konsultationstermin früher als üblich zu vereinbaren oder ihn rascher als sonst an einen Spezialisten zu überweisen.

❑ Ja ❑ Nein

1. A. Was ist Ihrer Ansicht nach die zutreffendste Diagnose? (Bitte nur eine Antwort ankreuzen.)

Meine zutreffendste Diagnose ist..........................

Es gibt mehrere mögliche Diagnosen; zum jetzigen Zeitpunkt kann ich keine wählen.

B. Und welche Diagnose bestimmt Ihren Behandlungsplan?.........................

1. Wie sicher sind Sie sich bei der Diagnose, die Sie bei Frage 8b als ausschlaggebend für Ihren Behandlungsplan angegeben haben? ____%
2. Bitte beschreiben Sie Ihr Bauchgefühl am Ende des Beratungsgesprächs:

❑ Hier stimmt etwas nicht.

❑ Alles passt zusammen.

❑ Kann ich unmöglich sagen, oder trifft nicht zu.
