# Supplementary material for: The linguistic validation of the gut feelings questionnaire in three European languages
Source: BMC Fam Pract. 2017 Apr 20;18:54. doi: 10.1186/s12875-017-0626-0 (PMC5437565; doi:10.1186/s12875-017-0626-0)
Supplement: Supplementary file 3 — GFQ Polish Version. The Polish version of the Gut Feeling Questionnaire. (DOCX 29 kb) [file 12875_2017_626_MOESM3_ESM.docx]

Całkowicie zgadzam się

Nie zgadzam się

Nie mam zdania

Zgadzam się

Zdecydowanie się zgadzam

1. Jestem pewny co do mojego planu postępowania i/lub wyników: wszystko zgadza się.. ❑ ❑ ❑ ❑ ❑
2. Jestem zaniepokojony stanem zdrowia tego

pacjenta: coś tu się nie zgadza. ❑ ❑ ❑ ❑ ❑

1. W tym konkretnym przypadku sformułuję tymczasowe

hipotezy z potencjalnie istotnymi wynikami i porównam je. ❑ ❑ ❑ ❑ ❑

1. Mam niejasne przeczucie ponieważ martwią mnie

potencjalnie niekorzystne wyniki.. ❑ ❑ ❑ ❑ ❑

1. Ten przypadek wymaga szczególnego postępowania aby

zapobiec dalszym poważnym problemom zdrowotnym. .. ❑ ❑ ❑ ❑ ❑

1. Jaki rodzaj postępowania wybrałeś? (zaznacz jedną odpowiedź)

❑ Poczekam i zobaczę jak się sytuacja rozwinie.

❑ Nie podejmę jeszcze działania, ale umówię się z pacjentem na wizytę kontrolną w gabinecie lub na konsultację telefoniczną.

❑ Zlecę dalsze badania (badania laboratoryjne, RTG, itd.).

❑ Zlecę dalsze badania a w międzyczasie rozpocznę leczenie (leki lub inny rodzaj postępowania).

❑ Rozpocznę leczenie bez umawiania.

❑ Rozpocznę leczenie i umówię pacjenta na wizyty kontrolne w gabinecie lub na konsultację telefoniczną.

❑ Skieruję pacjenta gdzieś indziej.

1. Sytuacja pacjenta daje mi podstawy aby umówić go na wizytę kontrolną wcześniej niż zwykle lub skierować jego lub ją do specjalisty szybciej niż zwykle.

❑ Tak ❑ Nie

1. A. Jaka według Ciebie diagnoza jest najbardziej prawdopodobna? (Proszę zaznaczyć jedną odpowiedź).

- Najbardziej prawdopodobną diagnozą według mnie jest….....................

- Istnieje kilka możliwych rozpoznań; nie jestem w stanie w tym momencie wybrać jednego z nich.

B. Która diagnoza w takim razie zdecyduje o Twoim postępowaniu............................

1. Na ile jesteś pewny tej diagnozy, którą wskazałeś w punkcie 8b jako decydującą o Twoim postępowaniu?____%
2. Proszę określić jaki rodzaj przeczucia występuje u Ciebie pod koniec konsultacji:

❑ Wydaje się, że nie wszystko tutaj jest w porządku.

❑ Wszystko pasuje.

❑ Nie da się stwierdzić albo nie dotyczy.
