# Supplementary material for: The linguistic validation of the gut feelings questionnaire in three European languages
Source: BMC Fam Pract. 2017 Apr 20;18:54. doi: 10.1186/s12875-017-0626-0 (PMC5437565; doi:10.1186/s12875-017-0626-0)
Supplement: Supplementary file 4 — GFQ English version. The English version of the Gut Feeling Questionnaire. (DOCX 27 kb) [file 12875_2017_626_MOESM4_ESM.docx]

Completely Disagree

Disagree

Neutral

Agree

Completely Agree

1. I feel confident about my management plan and/or about the outcome: it all adds up. ❑ ❑ ❑ ❑ ❑
2. I am concerned about this patient’s state of health:

something does not add up here. ❑ ❑ ❑ ❑ ❑

1. In this particular case, I will formulate provisional

hypotheses with potentially serious outcomes and weigh them

against each other. ❑ ❑ ❑ ❑ ❑

1. I have an uneasy feeling because I am worried about

potentially unfavourable outcomes. ❑ ❑ ❑ ❑ ❑

1. This case requires specific management to prevent any

further serious health problems. ❑ ❑ ❑ ❑ ❑

1. What course of action have you chosen? (Please tick one answer.) I will:

❑ Wait and see.

❑ Not yet take action, but will invite the patient for a follow‐up appointment either

face‐to‐face or by phone.

❑ Arrange further testing (laboratory tests, X‐rays, etc.).

❑ Arrange further testing, and in the meantime, I will start treatment (medicinal

or other).

❑ Start treatment, but will not arrange a follow‐up.

❑ Start treatment and will invite the patient for a follow‐up appointment either

face‐to‐face or by phone.

❑ Refer the patient.

1. This patient’s situation gives me reason to arrange a follow‐up visit sooner

than usual or to refer him or her more quickly than usual to a specialist.

❑ Yes ❑ No

1. A. What do you consider to be the most likely diagnosis? (Please tick one answer.)

My most likely diagnosis is ………………………………………………….

There are several possible diagnoses; I am unable to choose one at this moment.

…………………………………………………………

B. And which diagnosis will determine your management?

………………………………

1. How confident are you in the diagnosis that you indicated under 8b as determining your

management? ____%

1. Please indicate what kind of gut feeling you have at the end of the consultation:

❑ Something is wrong with this picture.

❑ Everything fits.

❑ Impossible to say, or not applicable.……………………
